# Antibiotic Consumption in Vanuatu before and during the COVID-19 Pandemic, 2018 to 2021: An Interrupted Time Series Analysis

**DOI:** 10.3390/tropicalmed8010023

**Published:** 2022-12-27

**Authors:** Nicola D. Foxlee, Amsaline Lui, Agnes Mathias, Nicola Townell, Colleen L. Lau

**Affiliations:** 1 Department of Global Health, National Centre for Epidemiology and Population Health, Australian National University, Canberra, ACT 2600, Australia; 2 Dispensary, Vila Central Hospital, Ministry of Health, Port Vila 9009, Private Mail Bag, Vanuatu; 3 Curative Services, Vila Central Hospital, Ministry of Health, Port Vila 9009, Private Mail Bag, Vanuatu; 4 Hospital in the Home, Sunshine Coast University Hospital, Birtinya, QLD 4575, Australia; 5 School of Public Health, Faculty of Medicine, University of Queensland, Brisbane, QLD 4006, Australia

**Keywords:** ATC/DDD classification, AWaRe, antibiotic consumption, COVID-19 pandemic, Vanuatu

## Abstract

The study objectives were to examine antibiotic consumption at Vila Central Hospital (VCH), Vanuatu between January 2018 and December 2021 and the influence of the COVID-19 pandemic on antibiotic consumption during this period. Data on antibiotic usage were obtained from the Pharmacy database. We used the WHO’s Anatomical Therapeutic Classification/Defined Daily Dose (ATC/DDD) index, VCH’s inpatient bed numbers and the hospital’s catchment population to calculate monthly antibiotic consumption. The results were expressed as DDDs per 100 bed days for inpatients (DBDs) and DDDs per 1000 inhabitants per day for outpatients (DIDs). Interrupted time series (ITS) was used to assess the influence of COVID-19 by comparing data before (January 2018 to January 2020) and during (February 2020 to December 2021) the pandemic. Ten antibiotics were examined. In total, 226 DBDs and 266 DBDs were consumed before and during COVID-19 by inpatients, respectively with mean monthly consumption being significantly greater during COVID-19 than before the pandemic (2.66 (*p* = 0.009, 95% CI 0.71; 4.61)). Whilst outpatients consumed 102 DIDs and 92 DIDs before and during the pandemic, respectively, the difference was not statistically significant. Findings also indicated that outpatients consumed a significantly lower quantity of Watch antibiotics during COVID-19 than before the pandemic (0.066 (*p* = 0.002, 95% CI 0.03; 0.11)). The immediate impact of COVID-19 caused a reduction in both inpatient and outpatient mean monthly consumption by approximately 5% and 16%, respectively, and this was followed by an approximate 1% monthly increase until the end of the study. By mid-2021, consumption had returned to pre-pandemic levels.

## 1. Introduction

The Coronavirus Disease 2019 (COVID-19) outbreak was declared a global pandemic by the World Health Organization in March 2020 [1]. According to the WHO, 650 million people have been infected with the virus and 6.5 million have died [2]. Health systems globally have struggled to keep pace with the pandemic consequently, other pressing public health concerns in particular, antimicrobial resistance (AMR) have received less attention [3]. Antimicrobial resistance remains a major threat to global public health [3]. It has been estimated that AMR caused 1.8 million deaths or approximately one-third of the mortality rate of COVID-19 in 2020 alone [3]. Research into the use of antimicrobials, including antibiotics during the COVID-19 pandemic suggests an increase in antibiotic consumption, particularly in low- and middle-income-countries (LMICs) [4,5,6,7,8]. Experts have raised concerns about a potential rise in AMR resulting from unwarranted antibiotic prescription in COVID-19 patients [1,5,9]. This may be exacerbated in LMICs because of their already stressed health systems [9].

All member countries of the World Health Assembly have either implemented their National Action Plan (NAP) to combat AMR or are in the process of developing a plan based on objectives of the Global Action Plan on Antimicrobial Resistance (GAP) [10]. Surveillance and research (objective two) and, optimizing the use of antimicrobials through the provision of antimicrobial stewardship programs (ASPs; objective four) are important and interrelated components of the plan [11,12]. Monitoring and analysis of antibiotic consumption is critical for understanding AMR and informing ASPs [11,12]. To support ASPs, WHO developed the Access Watch and Reserve (AWaRe) classification of antibiotics in 2017 [13]. Access antibiotics have a lower potential for developing resistance than those in other groups and are effective against a wide range of infections. Most antibiotics in the Watch group are listed amongst the Critically Important Antimicrobials for Human Medicine and have a high potential for resistance [14]. The Reserve antibiotics are medicines of last resort to be used for infections caused by multi-drug-resistant organisms [13].

WHO provides an international standardised unit of measurement for antibiotic use [10]. The defined daily dose (DDD) is the assumed average maintenance dose per day for a medicine used for its main indication in adults [15,16]. Research has shown that the number of DDDs decreased during the COVID-19 pandemic in some countries [17,18,19], but increased in others [6,7,8,20]. A study from Jordan (2021), reported a reduction of 5.5% in antibiotic consumption in 2020 relative to 2019 [17]. A systematic review and meta-analysis in 2021 covering 13 countries found overall prevalence of antimicrobial consumption in patients with COVID-19 in hospital or secondary health-care settings to be 68% (95% CI 60%; 75%) [5]. Subgroup analysis in studies from high-income-countries (HICs) (*n* = 19) revealed the pooled proportion of antimicrobial use to be 58% (95% CI:48%; 67%) compared with 89% (95% CI: 82%; 94%) in lower-middle-income-countries countries (LMICs, *n* = 3) and upper-middle-income-countries (UMICs, *n* = 5) combined [5]. The authors point out that better supported health systems in HICs may explain this difference [5].

Inappropriate prescribing due to misdiagnosis and under- or over-prescribing can contribute to the emergence of AMR [1]. Uncertainty regarding how best to treat COVID-19 patients when therapeutic protocols were unavailable, the difficulty in differentiating between a viral and bacterial co-infection or superinfection [9], the overwhelming number of patients presenting for treatment, shortages of trained staff and disrupted supply chains may have severely challenged health worker decisions regarding antibiotic prescribing during the pandemic. Health systems in LMICs) are faced with logistical challenges that limit their response to natural or man-made upheavals and therefore, LLMICs were impacted by COVID-19 to a far greater extent [9].

The Pacific Island Countries and Territories (PICTs) are all LMICs. With assistance from international partners some PICTs remained almost COVID-free until the end of 2021 when 15 of 22 PICTs reported either no cases or less than 11 cases since the start of the pandemic [21]. At the time this article was written, this included Vanuatu which had reported just seven cases in returned travellers who were in isolation within the quarantine system and no reported community transmission [22]. Even though Vanuatu had experienced few COVID-19 cases prior to the end of 2021, pandemic preparedness and response activities, such as the nation-wide safe COVID-19 messaging and restrictions regarding social interactions and travel may have influenced the use of hospital services and therefore, the consumption of antibiotics [23,24,25].

There has been limited research into antibiotic consumption in PICTs and as far as we are aware, research into this topic has not been conducted in Vanuatu. Therefore, the objectives of this study were to: (a) examine antibiotic consumption and AWaRe prescribing at Vila Central Hospital (VCH) before the pandemic (January 2018 to January 2020) and during the initial stages of COVID-19 (February 2020 to December 2021); and (b) assess whether COVID-19 influenced antibiotic consumption at VCH by comparing usage between the two time periods.

## 2. Materials and Methods

### 2.1. Study Setting

Vanuatu is a small island nation with a population of about 300,000 located in the southwest Pacific [26]. The country is divided into six Provinces and two Health Directorates: Southern and Northern. Vanuatu’s main referral hospital, VCH is in the capital, Port Vila. VCH serves the Southern Health Directorate, whilst the smaller Northern Provincial Hospital (NPH) serves the Northern Health Directorate. Both VCH and NPH are supported by several provincial hospitals, 34 health centres, 94 dispensaries and numerous aid posts. VCH’s catchment area covers the Province of Shefa which has a current population of 107,337 [26]. As the main referral hospital, VCH also accepts patients transferred or referred from other health services in Vanuatu. The hospital has 230 beds and delivers the following outpatient services: accident and emergency; general and children’s outpatient; surgery and medicine outpatient; oral health; ear, nose and throat care; eye care; mental health (referred to as mind care); maternity and family planning; and physiotherapy.

### 2.2. The COVID-19 Pandemic

Vanuatu’s response to the pandemic was immediate. By the end of January 2020, the country’s COVID-19 Health Sector Preparedness and Response Plan had been developed. The plan prioritises actions according to situational changes and number of COVID-19 cases: no cases; one or more cases and community transmission [27]. Training in COVID-19 preparedness commenced in February 2020. Vanuatu is a member of the Pacific Public Health Surveillance Network and prior to the pandemic had been reporting weekly from 11 sites on five core syndromes: acute fever and rash, prolonged fever, influenza-like illness, watery diarrhea and dengue-like illnesses [27,28]. This was increased by 13 additional sites by the end of March 2020 [27]. On 26th March 2020, the first suspected case of COVID-19 was reported, and Vanuatu closed its borders. The border remained closed throughout the remainder of our study period [29].

February 2020 was selected as the nominal start of the COVID-19 period in Vanuatu (beginning of the “intervention”) as this was considered the time when health workers and the community generally would be experiencing raised awareness of the pandemic. Therefore, for the purpose of this study, data from January 2018 to January 2020 (inclusive) were considered representative of before the pandemic. Consumption patterns and data collected from February 2020 to December 2021 (inclusive) were used to indicate antibiotic use during the early stages of the pandemic. More information can be found in Table S1 (Supplementary File) which lists the major activities and events that occurred during the pandemic period in Vanuatu.

### 2.3. Data Collection

The Vanuatu Ministry of Health maintains an Essential Medicines List (EML) which is regularly updated [30]. The antibiotics included in this study were those listed on the EML. The monthly quantities of each antibiotic prescribed for oral consumption were drawn from VCH’s pharmacy database. The data included antibiotics dispensed to outpatients and for inpatients for the period January 2018 to December 2021. The WHO’s Anatomical Therapeutic Chemical/DDD (ATC/DDD) index was interrogated to obtain the ATC code and DDD for each antibiotic [31]. The DDD is reported as DDDs per 1000 inhabitants per day for outpatient use (DIDs) and DDDs per 100 bed days (DBDs) for inpatient usage [32]. For the purposes of this study, we used only medicines classified into ATC code J01 indicating antibacterials for systemic use [11]. Our calculations used VCH’s annual catchment populations; these are provided in Table S2 in the Supplementary File.

### 2.4. Data Analysis

We used an interrupted time series (ITS) design which employed segmented regression analysis to assess whether COVID-19 had changed outpatient antibiotic consumption at VCH [33]. The ITS design was chosen because it is a more robust alternative to a before and after study [34]. 

The data for our ITS analysis were collected at monthly intervals between January 2018 and December 2021 which generated 25 time points before and 23 time points during the pandemic. The data included the following variables: DDDs, month, year, time (elapsed time since the start of the study) before or during the intervention (the COVID-19 pandemic) and the catchment populations [26]. We hypothesized that, if the pandemic affected antibiotic consumption, there would be a level change in the slope indicating either a decrease or increase in consumption. We used a generalised linear regression model with a Poisson distribution in our ITS design. Our unadjusted regression models included the counterfactual scenario whereby had COVID-19 not occurred, the trend in antibiotic use before the pandemic would have continued unchanged until the end of the study. Our final models were adjusted for dispersion (variability within the dataset) and seasonality was controlled for by fitting Fourier terms [35]. Autocorrelation was assessed during model checking and Akaike and Bayesian information criteria were used to measure model fit [33,36]. All analyses were conducted using Stata version 15 (Stata Corp, College Station, TX, USA). Two-sample t-tests were used for comparison and a *p*-value of ≤0.05 was considered statistically significant.

## 3. Results

### 3.1. Consumption before and during COVID-19

There were 10 antibiotics consumed by both inpatients and outpatients during the study period. All were administered orally and classified according to the ATC/DDD system as antibiotics for systemic use (JO1). Table 1 provides details of the antibiotics, ATC codes, the DDD unit of measurement and AWaRe classification.

### 3.2. Inpatient Consumption

Inpatients consumed a total of 226.9 DBDs (15,661 DDDs) during the 25 months before the pandemic and 266.8 DBDs (18,411 DDDs) during the first 23 months of COVID-19. There was a significant difference between mean monthly consumption before (9.07 DBDs) when compared with during the pandemic (11.6 DBDs) of 2.66 (*p* = 0.009, 95% CI 0.71; 4.61). The penicillins were the most prescribed class of antibiotic for inpatients and made up approximately 70% before and during COVID-19. Within this antibiotic class, cloxacillin was the most frequently consumed antibiotic making up 37% during both periods. Metronidazole also made up a high proportion of overall consumption at approximately 20% throughout the study period.

### 3.3. Outpatient Consumption

A total of 102 DIDs (314,004 DDDs) were consumed before and 92.7 DIDs (295,508 DDDs) during the pandemic. It can be seen from Table 2 that whilst there was a slight difference in the mean monthly consumption of DIDs during the two periods, the difference was not significant. The penicillin made up almost 80% of outpatient consumption during both periods. Amoxicillin followed by phenoxymethylpenicillin comprised 40% and 16% of the total, respectively, and metronidazole accounted for approximately 12% of total prescribing.

Further information can be found in Table 2 which provides the amount prescribed in DDDs, DBDs and DIDs before and during COVID-19. The line graphs in Figure S1a,b (Supplementary File) compare the difference in mean monthly consumption of DDDs for inpatients (Figure S1a and outpatients Figure S1b) during the two periods: January 2018 to January 2020 and February 2020 and December 2021. Table 3 lists the antibiotics by type with the percentage of total consumption before and during COVID-19 by inpatients and outpatients.

All antibiotics included in the study belonged to either the AWaRe Watch or Access class. The Watch class AWaRe antibiotics used with both inpatients and outpatients at VCH included the two macrolides (azithromycin and erythromycin) and the fluoroquinolone, ciprofloxacin.

Inpatient consumption of Watch antibiotics made up ≤7% (16.2 DBDs) and ≤6% (15.8 DBDs) of the total before and during the pandemic, respectively. There was no significant difference between the mean monthly amounts consumed during the two periods. Prescribing of Access antibiotics accounted for the remaining 93% and 94%, respectively.

Outpatient consumption of Watch class antibiotics made up ≤7% of total consumption before the pandemic and ≤5% during the pandemic. However, there was a significant difference in mean monthly consumption of Watch antibiotics by outpatients before (6.9 DIDs) and during COVID-19 (4.8 DIDs) of 0.066 (*p* = 0.002, 95% CI 0.03; 0.11), indicating a decrease in prescribing of these antibiotics in the later period. Additional information can be found in Table 4, which lists the antibiotics and the amounts consumed before and during the pandemic. 

### 3.4. Influence of the COVID-19 Pandemic on Antibiotic Consumption

**Figure 1:**
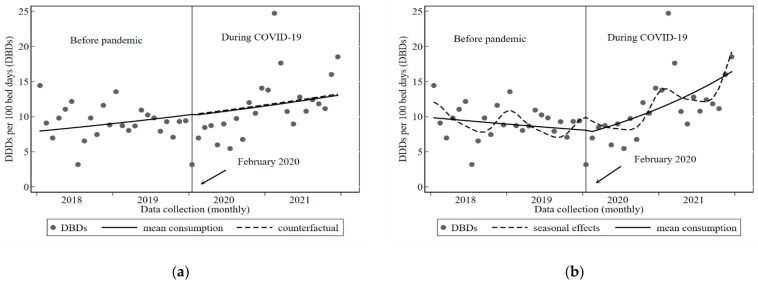
Inpatient antibiotic consumption at Vila Central Hospital, Vanuatu before and during COVID-19, 2018 to 2021: (**a**) Unadjusted model showing counterfactual; (**b**) Final model showing seasonal effects.

**Figure 2:**
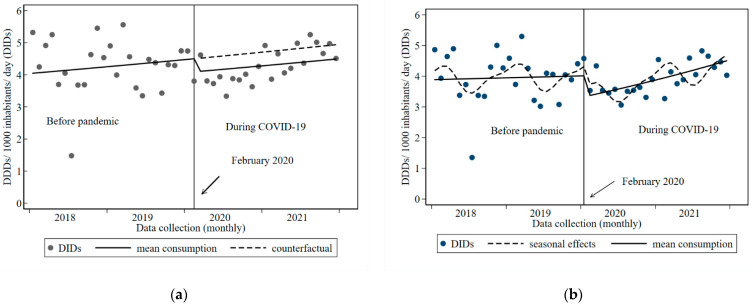
Outpatient antibiotic consumption at Vila Central Hospital, Vanuatu before and during COVID-19, 2018 to 2021: (**a**) Unadjusted model showing counterfactual; (**b**) Final model showing seasonal effects.

We used ITS analysis to assess whether COVID-19 had changed inpatient or outpatient antibiotic prescribing at VCH. Figure 1a,b and Figure 2a,b show the results of the unadjusted and final regression models for inpatient and outpatient antibiotic prescribing from January 2018 to December 2021, respectively. The models in Figure 1a and Figure 2a also include the counterfactual scenarios, whilst the final models in Figure 1b and Figure 2b include the seasonal effects. Additional information can be found in Figure S1a,b in the Supplementary File, which show the difference in mean monthly consumption for inpatients and outpatients before and during COVID-19.

Inpatient consumption is shown to have been decreasing pre-pandemic in the final adjusted regression model as indicated by the direction of the slope (Figure 1b). The impact of COVID-19 was a slight decrease (5%) in monthly DBDs (IRR 0.94, *p* = 0.76, 95% CI 0.65) followed by a significant month-on-month increase (IRR 1.04, *p* = 0001, 95% CI 1.02; 1.07) which continued until the end of the study.

Outpatient antibiotic consumption during the period before the pandemic was stable. The immediate impact of COVID-19 was a 16% decrease in monthly DIDs (IRR 0.83, *p* = 0.074 95% CI 0.68; 1.01) indicated by a step change in the slope after the beginning of February 2020. This was followed by an increase in antibiotic consumption by approximately 1% each month (IRR 1.01, *p* = 0.098, 95%CI 0.99; 1.02) during COVID-19 until the end of the study. By April 2021 consumption had returned to pre-pandemic levels (Figure 2a,b). Additional information can be found in Table S3a,b in the Supplementary File, which provide the results of the final regression models for inpatient and outpatient antibiotic consumption before and during COVID-19.

## 4. Discussion

The findings of our examination of antibiotic prescription before and during the early period of COVID-19 revealed slight differences in quantities consumed for both inpatients and outpatients. There was a significant increase in DBDs consumed by inpatients during COVID-19 when compared with before the pandemic and outpatients consumed slightly less DIDs during COVID-19 when compared with before. Though the difference was not statistically significant. Similar findings have been reported in research from other LMICs which compared consumption during 2019 (pre-pandemic) with 2020 (early COVID-19) [6,7,17]. Hospital inpatient studies from Egypt and Pakistan both report increases in the quantity of DBDs consumed during 2020 of 16.3% and 37.4%, respectively, when compared with 2019 consistent with findings from VCH [6,7]. A Jordanian study found an overall decrease in DIDs of 5.5% during 2020 compared with 2019, also consistent with results from Vanuatu [17]. The increase in DBDs in Vanuatu during early COVID-19 may be explained by a reliance on empiric therapy due to the lack of local therapeutic guidelines and microbiology laboratory capacity as well as deficiencies in infection prevention and control strategies [9].

The penicillins were the most frequently prescribed class of antibiotic, comprising approximately three-quarters of total consumption by both inpatients and outpatients throughout the study period. Cloxacillin and amoxicillin were the antibiotics of choice for inpatients and outpatients, respectively, accounting for almost half of the total antibiotics consumed both before and during COVID-19. These findings are not surprising as Vanuatu’s EML, like many other LMICs is limited. A WHO global surveillance report (65 countries) found amoxicillin to be one of the highest consumed antibiotics in most countries in 2018 [37]. We were unable to locate studies from other LMICs reporting findings about use of the penicillins during the pandemic consistent with our results. Studies reporting antibiotic consumption from other PICTs are scarce making it difficult to make comparisons with similar settings. The fact that collecting consumption data is resource-intensive and expensive may explain this scarcity. One other study from the PICTs was identified. An older study from Samoa reported total antibiotic consumption in 2007 to be 37.3 DIDs (mean monthly DIDs = 3.1) with the penicillins comprising 63% [38]. Whilst the results of this study cannot be compared with those from VCH as the data sources differ, they do give an indication of consumption in another PICT. The absence of similar studies from PICTs highlights the need for further research into antibiotic utilisation in PICTs.

The decrease in DIDs consumed by outpatients during COVID-19 when compared with before the pandemic may have been associated with the immediate 16% reduction in prescribing at the beginning of the pandemic (February 2020). Monthly consumption remained below the level experienced before the pandemic until mid-2021 when prescribing returned to before pandemic levels. The aim of the AWaRe classification is to reduce the use of Reserve and Watch group antibiotics and increase use of antibiotics in the Access group where possible [39]. Reserve group antibiotics were not listed on Vanuatu’s limited EML This is possibly because access was financially out of reach. Throughout the study period, prescribing of Watch antibiotics remained at ≥7% for both in- and outpatients. The goal of WHO’s AWaRe project is for all countries to have improved prescription practices so that at least 60% of all antibiotics used come from the Access group [39]. Notably, 94% of the prescribing for both in- and outpatients was related to Access antibiotics throughout the study period.

There are several possible explanations for the decrease in antibiotic usage during the early COVID-19 period. At the time Vanuatu’s preparedness and response plan was made public, health worker training commenced, and infection prevention and control practices were intensified [22,27]. The nation-wide COVID-19 safe messaging campaign, restrictions on social gatherings and travel may have resulted in minimising social contacts and the likelihood of transmission and consequently, the need for health services. It is also possible that public fear (the hospital being seen as a focus of disease) may have kept patients away from presenting to the hospital. In association, the diversion of core health personnel from normal duties to manage COVID-19 related activities may have caused disruptions in health services generally, thus reducing the amount of antibiotic consumption [29].

In May 2021 Vanuatu received its first shipment of Astra Zeneca COVID-19 vaccines and in June the vaccination programme was launched in Shefa and in other Provinces in the months following [22]. It may be that by mid-2021 due to limited cases of COVID-19, no reported cases of community transmission, people were potentially feeling more confident and returning to normal social engagements. This may have paved the way for an increase in outpatient clinic attendance, infectious disease transmission and a need for antibiotic treatment. This may provide an explanation for the continued increase in antibiotic consumption.

### 4.1. Strengths

To the best of our knowledge, this is the first published study examining antibiotic consumption in Vanuatu. The use of the ITS design to measure the changes in antibiotic consumption before and during the COVID-19 pandemic is a strength of the study. This method can provide a high degree of internal validity [34]. Our model included 48 monthly data points, accounted for dispersion, adjusted for seasonal effects and assessed autocorrelation. We estimated the change in level and slope and included the counterfactual scenario.

### 4.2. Limitations

In our study antibiotic consumption by inpatients was limited to antibiotics consumed orally. Whilst it was suggested (word of mouth) that annual inpatient consumption of parenteral antibiotics amounted to approximately 30% of the total, this has not been substantiated. The authors are confident that the quantity of antibiotics consumed was the amount prescribed as, unlike some other LMICs antibiotics are not available without a prescription in Vanuatu.

Our calculations of DDDs per 100 bed days assumed full bed occupancy, as the actual rates were not available to us. Because our study relates only to VCH, the major referral hospital in Vanuatu and VCH’s catchment populations, our results may not reflect other health settings in Vanuatu.

### 4.3. Future Directions

Monitoring and analysis of antibiotic consumption data is key to understanding AMR [40]. The WHO has proposed ASPs as a key strategy to curb AMR [7]. Research into antibiotic consumption can inform ASPs. The results can be used to assess ASP strategies implemented to influence prescribing and may provide insight into the development of AMR. Research conducted longitudinally can be used to track trends and make comparisons with other healthcare settings nationally and regionally [40].

It is recommended that regular surveillance and analysis of dispensary data in Vanuatu be performed in all health care settings and results made available at all levels. Regular point prevalence surveys measuring the proportion of inpatients receiving an antibiotic can add to these results as well as provide insight into health care associated infections. Sharing results nationally will inform policies, regulations and ASP strategies to optimize antibiotic use (WHO). It is also recommended research into antibiotic prescription be conducted in other PICTs to inform regional strategies to combat AMR.

## Figures and Tables

**Table 1:** Antibiotics for systemic use (J01) consumed by inpatients and outpatients at VCH, Vanuatu 2018 to 2021 with ATC code, route of administration, DDD units in grams obtained from the ATC/DDD index 2022 and Access, Watch and Reserve classification.

Antibiotic	Class	ATC Code	Route	DDD (g)	AWaRe Group
Amoxicillin	Penicillins	J01CA04	0ral	1.5	Access
Amoxicillin Clavulanate	Beta-lactam/beta-lactamase inhibitor	J01CR02	0ral	0.625	Access
Azithromycin	Macrolides	J01FA10	0ral	0.3	Watch
Ciprofloxacin	Fluoroquinolones	J01MA02	0ral	1	Watch
Cloxacillin	Penicillins	J01CF02	0ral	2	Access
Doxycycline	Tetracyclines	J01AA02	0ral	0.1	Access
Erythromycin	Macrolides	J01FA01	0ral	1	Watch
Metronidazole	Imidazole	J01XD01	0ral	1.5	Access
Phenoxymethylpenicillin	Penicillins	J01CE02	0ral	2	Access
Sulfamethoxazole/Trimethoprim	Sulphonamide/trimethoprim	J01EE01	0ral	4	Access

ATC = Anatomical Therapeutic Chemical classification; DDD = defined daily dose unit; VCH = Vila Central Hospital.

**Table 2:** Defined daily doses (DDDs) prescribed to inpatients and outpatients at Vila Central Hospital, Vanuatu before and during COVID-19: January 2018 to January 2020 and February 2020 and December 2021, respectively, with difference in mean monthly consumption before and during COVID-19.

Antibiotic Consumption	Before the Pandemic Jan 2018–Jan 2019	During COVID-19 Feb 2020–Dec 2021
	**Inpatient oral antibiotic consumption**	
Total DDDs	15,661	18,411
Total DBDs	226.97	266.82
Mean monthly DBDs	9.07	11.60
Difference between mean monthly DBDs before and during COVID-19	2.66 *p* = 0.009 (95% CI 0.71; 4.61)	
	**Outpatient oral antibiotic consumption**	
Total DDDs	314,004	295,508
Total DIDs	102.2	92.7
Mean monthly DIDs	4.09	4.03
Difference between mean monthly DIDs before and during COVID-19	0.063 *p* = 0.74 (95% CI−0.33, 0.46)	

DDD = defined daily dose; DBD = DDD/100 bed days; DID = DDD/1000 inhabitants/day.

**Table 3:** Antibiotics prescribed by type in defined daily doses (DDDs) by inpatients and outpatients at Vila Central Hospital, Vanuatu before and during COVID-19: January 2018 to January 2020 and February 2020 and December 2021, respectively, with percentage of total consumption and DDD unit of measurement.

Antibiotics Prescribed (DDD Unit)	Before the Pandemic: Jan 2019–Jan 2020	During COVID-19: Feb 2020–Dec 2021
**Inpatient DDDs/100 bed days (DBDs) (% total)**		
Amoxicillin (1.5 g)	46.2 (20.3)	53.9 (20.2)
Cloxacillin (2 g)	84.6 (37.2)	100.1 (37.5)
Penicillin V (2 g)	8 (3.5)	16.8 (6.3)
Amoxicillin/clavulanate (1.5 g)	17.6 (7.7)	23.8 (8.9)
Azithromycin (0.3 g)	2.9 (1.3)	2.7 (1)
Erythromycin (1 g)	3.2 (1.4)	2.8 (1)
Ciprofloxacin (1 g)	10.1 (4.4)	10.3 (3.9)
Doxycycline (0.1 g)	5.2 (2.3)	6.2 (2.3)
Sulfamethoxazole/Trimethoprim (4 g)	0.7 (0.3)	1.1 (0.4)
Metronidazole (1.5 g)	48.5 (21.4)	49.1 (18.4)
**Total DBDs per period**	**226.9 (100)**	**266.8 (100)**
**Outpatient DDDs/1000 inhabitants/day (DIDs) (% total)**		
Amoxicillin (1.5 g)	43.1 (42.2)	35.7 (38.5)
Cloxacillin (2 g)	14.1 (13.8)	14.2 (15.3)
Penicillin V (2 g)	13.8 (13.5)	15.9 (17.1)
Amoxicillin/ clavulanate (1.5 g)	6.7 (6.5)	5.5 (5.5)
Azithromycin (0.3 g)	2.3 (2.3)	2.4 (2.5)
Erythromycin (1 g)	1.02 (1.0)	0.5 (0.6)
Ciprofloxacin (1 g)	3.4 (3.3)	1.9 (2.1)
Doxycycline (0.1 g)	3.3 (3.2)	2.9 (3.2)
Sulfamethoxazole/Trimethoprim (4 g)	2.3 (2.2)	1.6 (1.7)
Metronidazole (1.5 g)	12.3 (12.1)	12.12 (13.1)
**Total DIDs per period**	**102.2 (100)**	**92.7 (100)**

**Table 4:** Antibiotic consumption by AWaRe classification for inpatients and outpatients at VCH, Vanuatu before and during COVID-19: January 2018 to January 2020 and February 2020 to December 2021, respectively, with percentage of total consumption according to AWaRe classification.

Antibiotic Prescription by Access Watch and Restrict Class (AWaRe)		
AWaRe Classification	Before the Pandemic: Jan 2019–Jan 2020	During COVID-19: Feb 2020–Dec 2021
	**Inpatient DDDs/100 bed days (DBDs) (% total)**	
**Watch class** DBDs	16.2 (7)	15.8 (6)
Difference between mean monthly Watch DBDs before and during COVID-19	0.041 *p* = 0.54 (95% CI−0.18; 0.09)	
**Access class** DBDs	210.7 (93)	251 (94)
Difference between mean monthly DBDs before and during COVID-19	0.75 *p* = 0.49 (95% CI−2.93; 1.43)	
	**Outpatient DDDs/1000 inhabitants/day (DIDs)** **(% total)**	
**Watch class** DIDs	6.9 (7)	4.8 (5)
Difference between mean monthly DIDs before and during COVID-19	0.066 *p* = 0.002 (95% CI 0.03; 0.11)	
**Access class** DIDs	95.5 (93)	87.9 (95)
Difference between mean monthly DIDs before and during COVID-19	0.008 *p* = 0.96 (95% CI−0.36; 0.38)	

## Data Availability

Availability of data is at the discretion of the Vanuatu Ministry of Health.

## Supplementary Materials

The following supporting information can be downloaded at: https://www.mdpi.com/article/10.3390/tropicalmed8010023/s1, Figure S1: Differences between mean monthly antibiotic consumption before and during COVID-19: January 2018 to January 2020 and February 2020 to December 2021, respectively; Table S1: Key activities and events during early COVID-19 in Vanuatu: 2020 and 2021; Table S2: Populations of Shefa: 2018 to 2021; Table S3a,b: Results of the final regression models for inpatient and outpatient consumption following adjustment with the interaction term providing inter-rate ratios, standard errors, estimates and confidence intervals for the covariates.
