# Supplementary material for: Antibiotic Consumption in Vanuatu before and during the COVID-19 Pandemic, 2018 to 2021: An Interrupted Time Series Analysis
Source: Trop Med Infect Dis. 2022 Dec 27;8(1):23. doi: 10.3390/tropicalmed8010023 (PMC9864063; doi:10.3390/tropicalmed8010023)
Supplement: Supplementary file 1 [file tropicalmed-08-00023-s001.zip › tropicalmed-1992003-supplementary.pdf]

Antibiotic consumption in Vanuatu before and during the COVID-19 pandemic (2018 to 2021): Supplementary Files: Figures S1(a) and (b); Tables S1, S2 and S3(a) and (b)

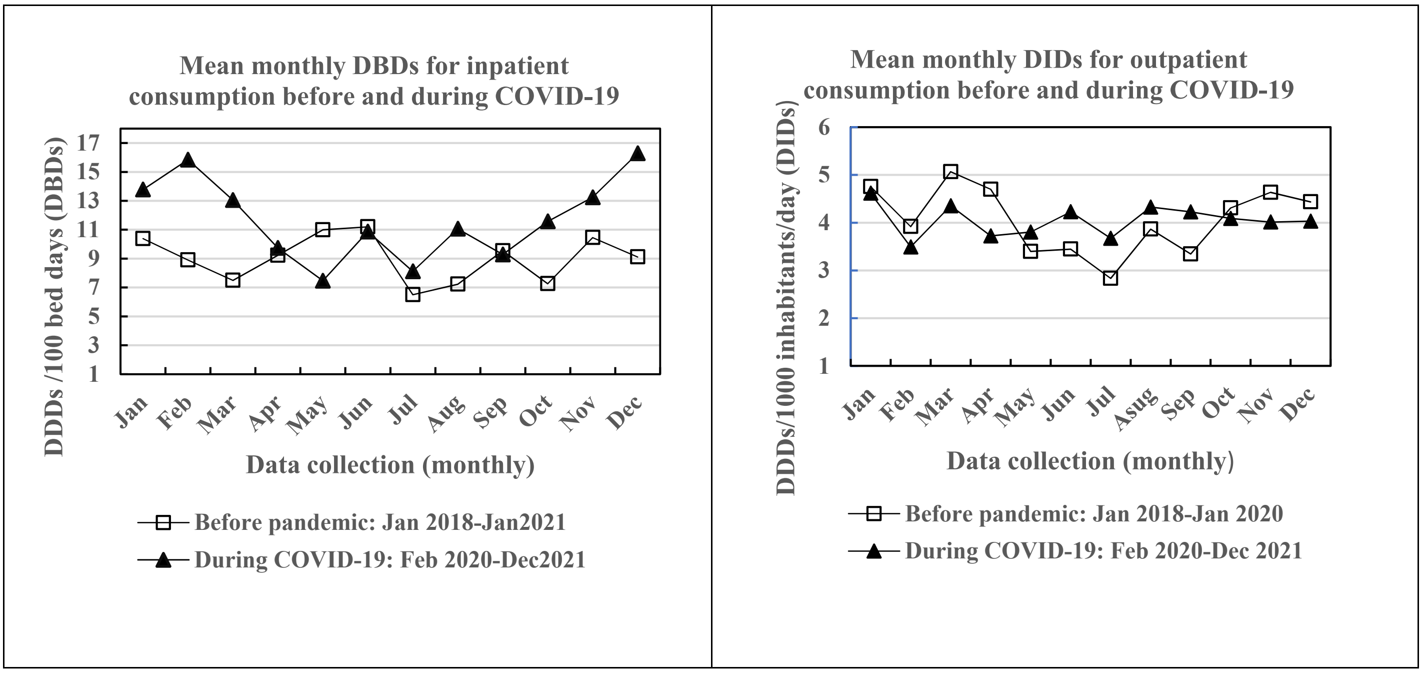

(a) (b)

Figure S1. Difference between mean monthly antibiotic consumption before and during COVID-19: Jan 2018 to Jan 2020 and Feb 2020 to Dec 2021, respectively DDDs; **(a)** showing inpatient usage in DBDs; **(b)** showing outpatient usage as DIDs.

Table S1. Key activities and events during early COVID-19 in Vanuatu: January 2020 to December 2021

Year	Activity Commenced or Event Occurred	Ongoing
**2020**		
Jan	COVID-19 taskforce formed; border restrictions commenced;	Y
23 Jan	Vanuatu preparedness and response plan released;	Y
Feb	Surveillance and contact tracing commenced; border restriction progressively enhanced; hospitals' preparation planning commences; health worker training begins;	Y
Mar	Nation-wide COVID safe messaging campaign: TV, radio, SM, print; telephone/email hotline and Ministry of Health COVID-19 website; Face book page for health promotion; community awareness activities; training of frontline workers;	Y
22 Mar	First suspected COVID-19 case; International border closed;	
Apr	Cyclone Harold (Samna, Panama, Malampa); state of emergency declared;	
May	Local SARS COV-2 testing established;	Y
Jun	Phase 1 repatriation of nationals and residents; hotel quarantine;	Y
Aug	Phase 2 repatriation of nationals and residents continues;	Y
Nov	15,064 cases & 135 deaths in PICTs	Y
**2021**		
22 Mar	2 COVID-19 cases identified in hotel quarantine - recovered;	
11 Apr	Deceased COVID-19 case on beach – foreign sailor;	
May	First COVID-19 Astra-Zeneca vaccines received;	Y
June	Vaccine rollout launched in Shefa with other Provinces in August, October & November;	Y
Nov	Two COVID-19 cases in hotel quarantine – recovered 7 COVID-19 cases for Vanuatu.	
Dec	160, 676 received first vaccine and 55,289 received second vaccination: June and end December, respectively;	Y
23 Dec	Efate and offshore islands alert level downgraded until further notice.	Y

Table S2. Populations of Shefa, Vila Central Hospital's catchment area during the study period: 2018 to 2021

Population of Shefa				
Year	2018	2019	2020	2021
population	107337	103270	105336	107337

Table S3 (a) and (b) Results from the final regression models for inpatients and outpatient consumption adjusted for seasonality with the interaction term, providing incidence rate ratios, standard errors, estimates and confidence intervals for the covariates; S3 (a) Inpatient consumption regression results for final model; S3 (b) Outpatient consumption regression results for final model.

S3 (a)

**Covariates**	**IRR**	**Std Err**	**P>|z|**	**95%CI**
covid	0.94454	0.1775	0.762	0.653; 1.365
Inter_covidtime	1.04256	0.0124	0.000	1.018; 1.067
time	0.99208	0.0085	0.253	0.973; 1.007
_cons	9.91794	1.2337	0.000	7.772; 12.656

S3 (b)

**Covariates**	**IRR**	**Std Err**	**P>|z|**	**95%CI**
covid	0.83482	0.0843	0.074	0.684; 1.017
Inter_covidtime	1.01104	0.0067	0.098	0.997; 1.024
time	1.00145	0.0046	0.753	0.992; 1.011
_cons	3.99311	0.2732	0.000	3.492; 4.566
